# Research on Multi-AUVs Data Acquisition System of Underwater Acoustic Communication Network

**DOI:** 10.3390/s22145090

**Published:** 2022-07-06

**Authors:** Chunxian Gao, Wenwen Hu, Keyu Chen

**Affiliations:** Key Laboratory of Underwater Acoustic Communication and Marine Information Technology, Ministry of Education, Xiamen University, Xiamen 361005, China; gaochunxian@xmu.edu.cn (C.G.); 23320191153268@stu.xmu.edu.cn (W.H.)

**Keywords:** underwater acoustic communication network, AUV, energy consumption, task assignment, path planning

## Abstract

In order to meet the needs of large-scale underwater operations, the underwater acoustic communication network emerged, marking a historic moment. At the same time, the development of artificial intelligence has promoted the application of intelligent underwater robots in large-scale underwater operations, and the research on related algorithms has been gradually promoted. Due to the complexity of underwater operations and the difficulty of replacing batteries, the energy efficiency of intelligent underwater robots is particularly important in multi-AUVs data acquisition systems. In view of the energy consumption of multi-AUVs data acquisition systems in water acoustic cluster networks, this paper proposed the AE (A*-Energy) algorithm for multi-AUVs task assignment and path planning. Through the simulation experiment, it was proved that the AE algorithm proposed in this paper can effectively reduce the energy consumption of multi-AUVs data acquisition systems and has good energy efficiency.

## 1. Introduction

With advances in onshore wireless communication technology, the focus of research has turned to the ocean. Today, the continuous expansion of marine technology has gradually made the deployment of underwater acoustic sensor networks (UWASNs) possible, and has also contributed to establishing various marine applications, such as military reconnaissance and surveillance, anti-submarine and patrol, marine mapping, marine resources exploration, diving support and other such applications [1,2].

Among them, the data acquisition system is an important example of the application of communication technology. In sensor networks, the large amount of data generated by sensors greatly influences the lifetime of the network [3]. To collect such a high amount of perceptual data in an energy-efficient manner, the whole data acquisition system needs to be redesigned using various algorithms. In [3], a distributed database algorithm was proposed by improving data storage and query. By classifying the current status of distributed data and query management techniques, the proposed algorithm is able to optimize the energy expenditure in the network and retrieve more accurate information. In [4], a WSN energy balance cluster routing algorithm based on a mobile agent (EBMA) was proposed. Based on the cluster structure of the cellular topology, the proposed algorithm considers the energy balance of the inter-cluster and intra-cluster environments, and can effectively balance the energy consumption of large-scale networks. In addition, redesign data acquisition systems based on AUV are also commonly used. As a new generation of intelligent underwater robots, AUV has the ability to search independently, explore and collect data with high precision, which allows underwater operators to remove the obstacles of artificial cables and be more flexible. However, a single AUV is unable to meet the high requirements of the hydroacoustic communication network due to its own limited power, energy, and communication range. Therefore, the multi-AUVs system with efficient coordination has been paid increasing attention by scientific research institutions in various countries [5,6]. Hence, it proves a challenging problem to determine how a group of AUVs can be reasonably assigned to visit their corresponding targets and avoid obstacles automatically in the underwater environment, while guaranteeing the minimum total consumption of AUVs [7,8].

Recently, the research on multi-AUVs collaborative algorithms has gradually matured. In [9], an improved algorithm based on the fast-stepping algorithm was proposed, which is based on the ocean current rules to support path planning in a dynamic marine environment through four-level risk strategies. Although the algorithm has the advantages of stronger regularity, it is also only applicable for special scenarios. In [10], a hybrid algorithm combining line-of-sight and artificial potential field methods was proposed. The algorithm corrects the heading through the distance threshold and reduces the heading computation, but ignores the effect of path distance. In [11], an improved algorithm for large-scale underwater operations based on the fast-stepping method was proposed. The algorithm introduces the mobility constraints of AUV, such as safety depth, fuel consumption and collision risk, which further adapt to the underwater acquisition operation of AUVs. In [12], a hybrid optimization method based on [11] for complex underwater environments was proposed. The algorithm also introduces obstacle constraints such as sea current and reef, which make the underwater obstacle avoidance of AUV smoother, reduce the path search time and reduce the time cost. In [13], an improved algorithm based on the market mechanism was proposed. By simulating the auction mechanism of “tendering- bidding-winning-signing” in the market, the task allocation of multiple AUVs was realized, and the effectiveness of the whole system was improved with the combination of rate indicators and the token ring network. However, the algorithm only focused on task assignment and did not provide a specific scheme for path planning. In [14], a quantum-behaved particle swarm optimization algorithm based on the traditional particle swarm optimization algorithm was proposed. The algorithm designs a novel global parameter to control its evolution. In multi-AUVs collaboration, the algorithm has few control parameters and strong convergence, but a lower search efficiency. In [15,16], a hybrid algorithm based on SOM (Self-Organizing Map) and velocity synthesis was proposed. The algorithm assigns the AUV through the winning neuron selection rule of the SOM algorithm and plans the path of the AUV through the speed synthesis algorithm under the interference of ocean currents. However, the algorithm is prone to rapid jumps and is unable to avoid obstacles. In [17], a BISOM (Biologically Inspired Self-Organizing Map) algorithm was proposed for the problems of [15,16]. The proposed algorithm introduces the BINN model to realize the efficient allocation and path planning of AUV, and solves the speed jump problem. However, it produces the problem of AUV repeat allocation and increases the total navigation distance of the multi-AUVs system. In [18], a novel BINN map algorithm was proposed to solve the problems of [17]. The algorithm redesigns the AUV assignment rules to solve the repeated distribution problems. However, the algorithm produces unnecessary paths in non-boundary regions and ignores the data acquisition and steering maneuver energy consumption of the AUV.

Aiming to address some of the above problems, a novel AE (A*-Energy) algorithm was proposed in this study for a multi-AUVs data acquisition system of an underwater acoustic communication network. The main contributions of the AE algorithm can be summarized as follows:

1. In the process of traditional AUV data acquisition, it is often necessary to move to the corresponding cluster head target and then conduct data acquisition. However, without considering timeliness, this mode has higher energy consumption. Therefore, the strategy adopted by the AE algorithm is that the AUV will replace the cluster head nodes to directly collect the data of the child nodes to further reduce the data transmission energy consumption of the cluster network;
2. Path planning: In addition to the shortest path, the mobile steering energy consumption of the AUV should also be considered to ensure that the AUV has a minimum angle of steering maneuver. In the AE algorithm, the path planning of AUV introduces the diagonal heuristic function and the steering cost to minimize the total energy consumption of the path planning. In order to meet the requirements of low energy consumption and allocation conflict avoidance of multi-AUVs systems, the new task allocation rules are formulated.

The rest of the paper is organized as follows: Section 2 introduces some background information about the model of the multi-AUVs data acquisition system. The grid map construction, task assignment and path planning based on the AE algorithm are presented in Section 3. The simulation results and comparative analysis are introduced in Section 4. Finally, conclusions are drawn in Section 5.

## 2. The Model of Multi-AUVs Data Acquisition System

**Figure 1:**
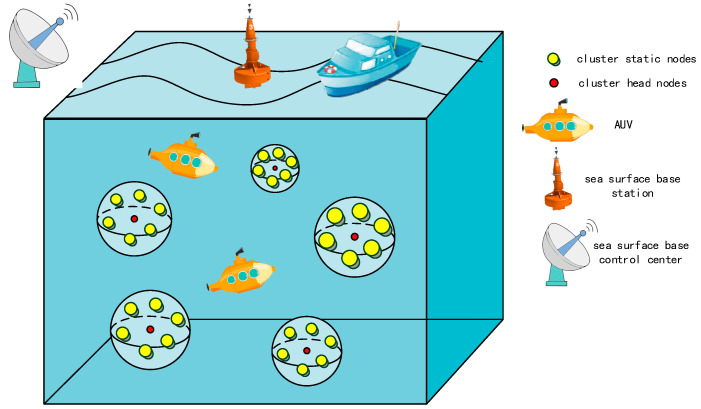
Multi-AUVs data acquisition system.

The application scenario of this paper is multi-target underwater data collection, with multiple objectives and a wide search range. As shown in Figure 1, the nodes deployed underwater can be divided into the following two categories: static nodes within the cluster and cluster head nodes. The whole multi-AUVs data acquisition system process is as follows: the static nodes in the cluster collect ocean data through the sensor device, and transmit the collected data to the cluster head node at the center of the cluster network. When the AUV arrives, the cluster head node transmits the collected data to the AUV through a single jump. The AUV travels next to the sea surface base station and sends the collected data to the receiving node of the sea surface base station. Finally, the sea surface base station transmits the data to the sea surface base control center for ocean data analysis. It should be noted that all nodes have the same computing and communication capabilities, and the AUV has more energy and data storage space than other nodes underwater, and is responsible for data collection in the cluster network. However, the AUV itself has limited energy during the search process. Due to the energy constraints, it is most important to consider minimizing the energy expenditure of the entire multi-AUVs data acquisition system when studying task assignment and path planning for multiple AUVs. Specifically, the energy consumption of AUVs is mainly related to the following three aspects: (1) Mobile energy consumption of multi-AUVs linear driving; (2) Mobile energy consumption brought by multi-AUVs steering; (3) Transmission energy consumption for multi-AUVs data acquisition. In this paper, we estimated the cost of a multi-AUVs data acquisition system using the total navigation distance from the start position to the final position, the total navigation angle, and the energy consumption.

Generally, the task allocation and path planning processes of a multiple AUVs data acquisition systems are as follows: The multi-AUVs system assigns the corresponding AUV to each task target according to the principle of minimum energy consumption, and plans the shortest barrier-free path between the AUV and the task target. The AUV travels to the task target under the dynamic constraints and starts to collect data after arrival. Once all the task objectives have been collected, the single data acquisition of the multi-AUVs data acquisition system is completed.

Assuming a set of nodes, $S=\left\{s_{1},s_{2},\ldots ,s_{n}\right\}$, they generate several clusters based on the clustering principle. Either cluster may be represented as $E=\left\{e_{1},e_{2},\ldots ,e_{j}\right\}$, where $e_{j}$ represents a child node in the cluster. The formed cluster does not set the cluster head node but uses AUV as the cluster head node, so it needs to reset the target position according to each cluster network. This paper adopts the principle of a geometric center to set the target position. Additionally, the sum of the distance from the child nodes in each cluster is minimal, which is generally the central position of the cluster network. The formed target clusters were considered as task clusters $C=\left\{c_{1},c_{2},\ldots ,c_{k}\right\},k<n$. The assigned AUV cluster is $R=\left\{R_{1},R_{2},\ldots ,R_{m}\right\},m\geq k$.

When an AUV is assigned a task, the path of the AUV to the target location is also determined simultaneously, and it can be determined that there are N points and N − 1 path segments in the path of the AUV. The energy expenditure of the AUV consists of propulsion energy and steering maneuvering energy [19]. The specific formula is as follows:

(1) $$E_{\mathrm{Tri}}=\sum_{i=1}^{N-1}E\left(L_{i}\right)=\sum_{i=1}^{N-1}\left(E_{\mathrm{ti}}+E_{\mathrm{turn}}\right),$$

where $E_{\mathrm{Tri}}$ indicates the total energy used by the AUV over the N − 1 path segments, linear propulsion energy is $E_{\mathrm{ti}}$ and the steering to maneuver the energy is $E_{\mathrm{turn}}$. $E_{\mathrm{ti}}$ is related to the path length of the AUV. $E_{\mathrm{turn}}$ is defined by:

(2) $$E_{\mathrm{turn}}=1-\cos\theta_{j,h}=1-\frac{L_{j}L_{h}}{\left\|L_{j}\|\|L_{h}\right\|}$$

where $\theta_{j,h}$ indicates the angle between path $L_{j}$ and path $L_{h}$ of two adjacent path segments. Considering the above description, the task assignment and path planning problems of AUVs can be summarized as follows:

(3) $$\rho_{\mathrm{opt}}=\arg\underset{}{\min}\;E_{\mathrm{Tri}}=\arg\underset{}{\min}\sum_{i=1}^{N-1}\left(E_{\mathrm{ti}}+E_{\mathrm{turn}}\right),$$

s.t.

(4) $$E_{\mathrm{Tri}}<\mathrm{energy},$$

(5) $$\underset{}{\min}\sum_{e_{j}\in c_{k}}r_{c_{k}},$$

(6) $$d_{c_{i}c_{j}}>d_{0},c_{i}c_{j}\in C,$$

where energy indicates the total energy of the AUV. Constraint (4) ensures that the initial energy of the AUV is sufficient for the assigned task. To reduce the energy consumption of data transmission within a clustered network, the sum of the target position $c_{k}$ to the child nodes $e_{j}$ in each cluster should be minimal. Additionally, the selection of the target location is constrained by (5). Constraint (6) ensures that the distance between target locations should be greater than the threshold distance for underwater data information transmission $d_{0}$, so that data transfer between target tasks does not interfere.

In conclusion, the optimization of the multi-AUVs system mainly includes the following two objectives: $E_{\mathrm{ti}}$ and $E_{\mathrm{turn}}$. $E_{\mathrm{ti}}$ can reduce the driving energy consumption by shortening the driving distance of the AUV. Additionally $E_{\mathrm{turn}}$, can reduce this by reducing the AUV steering maneuver angle. Therefore, the optimization of the energy consumption of a multi-AUVs system around these two aspects is the next issue to discuss.

In addition to the above AUV mobile energy consumption constraints, the transmission energy consumption of an AUV in the hydro acoustic communication cluster network also needs to be analyzed. The energy model of water acoustic channel data transmission mainly considers the noise disturbance caused by turbulence, shipping activity, thermal noise and wind in the ocean environment. Usually, the communication process of the underwater acoustic channels shows the path-loss scattering phenomenon due to the special nature of the underwater environment, such as the absorption of the medium and the scattering phenomenon of magnesium sulfate in seawater. In this study, the path loss was calculated based on the given distance d and frequency f [19], as follows:

(7) $$A\left(d,f\right)=A_{0}d^{k}\alpha {\left(f\right)}^{d},$$

where is A_0_ is a standardized constant and k is propagation loss. In addition, α(f) represents the absorption efficiency, which can be described according to *Thorp’s* relation [20]:

(8) $$10\log\alpha \left(f\right)=0.11\frac{f^{2}}{1+f^{2}}+44\frac{f^{2}}{4100+f^{2}}+2.75\times 10^{-4}f^{2}+0.003,$$

Generally, the energy consumption of underwater data transmission mainly consists of the energy consumption of acoustic communication and data transmission, which can be obtained from the following general energy consumption formula [21]:

(9) $$E_{\mathrm{ij}}=q_{\mathrm{sp}}+\alpha \left(f\right)d_{\mathrm{ij}}^{2}\;\;0<d_{\mathrm{ij}}<d_{0},$$

where $E_{\mathrm{ij}}$ stands for the energy consumption for node i to transmit one bit of information to another node j. $q_{\mathrm{sp}}$ is the energy consumption for data processing in the sending procedure. $d_{0}$ is the threshold distance to transmit data information. $q_{\mathrm{rp}}$ is the energy consumption for data processing in the sending procedure. So, the energy consumption formula for transmitting n bits of information between two nodes apart from d is as follows:

(10) $$E_{\mathrm{tx}}=n\times \left(q_{\mathrm{rp}}+q_{\mathrm{sp}}+\alpha \left(f\right)d^{2}\right)\;0<d<d_{0},$$

## 3. AE Algorithm

**Figure 2:**
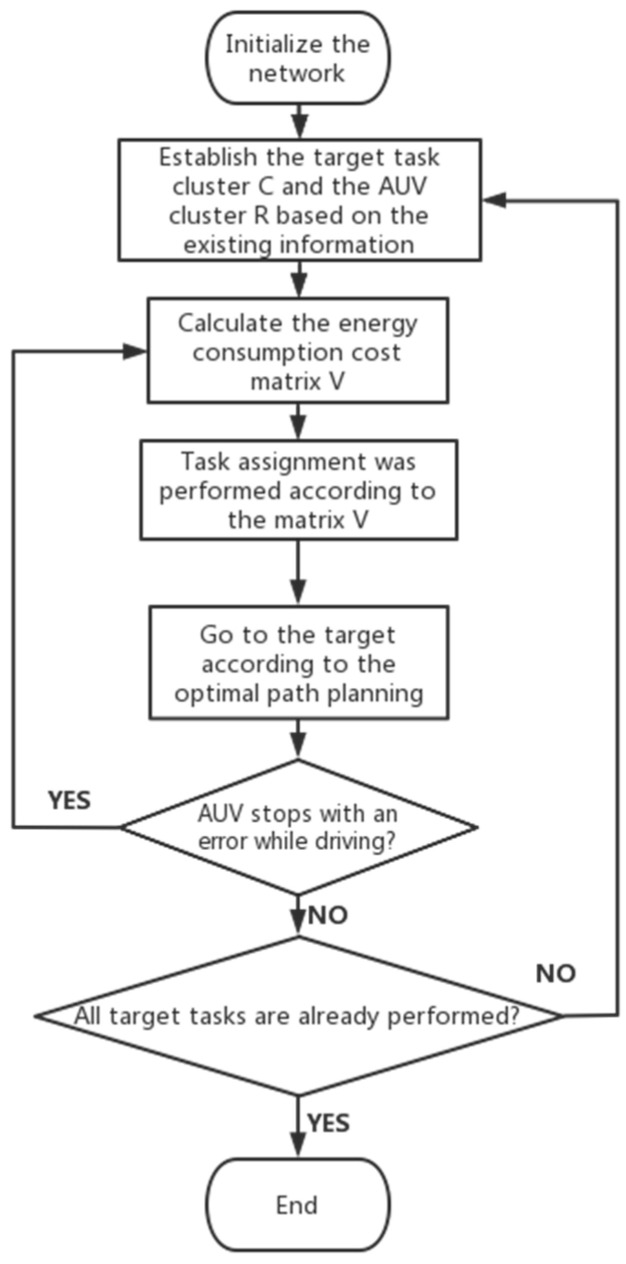
Flow chart of the AE algorithm.

This section presents task assignment and path planning methods for multi-AUVs systems. The main process of the multi-AUVs system is as follows: First, the underwater environment with a randomly distributed AUV, cluster network and obstacles is translated into a grid map. Second, the proposed AE algorithm performs task assignment and path planning for the AUV. Finally, the AUV performs the corresponding task. The proposed flowchart of the AE algorithm applied to multi-AUVs task assignment, path planning, and data acquisition is shown in Figure 2. First, during the network initialization process, the data model needs to be constructed based on the existing map environments, target clusters, AUV clusters, etc. Then, using the constraint formula and path planning algorithm, the energy consumption cost matrix V can be calculated and can be used to assign AUV tasks. Finally, the AUVs are assigned to perform their respective target tasks until all target tasks are completed. In addition, the AUV may stop due to an error during driving. In this case, the AUV retreats to the stage of the energy cost matrix calculation.

The path planning of the AE algorithm was mainly improved based on the traditional A* algorithm. The A* algorithm is a graph-based heuristic search algorithm that introduces the cost function to prevent the algorithm from falling into the local optimal solution [22] while retaining the Dijkstra algorithm path to search for the global optimal solution. Recently, due to its optimality and high efficiency, the A* algorithm has been widely used in the field of the algorithm and path planning. The A* algorithm is a heuristic algorithm with the following function:

(11) f(n)=g(n)+h(n),

where f(n) indicates the cost function from the beginning to the end of planning. h(n) indicates the cumulative estimation function that also needs to be generated from the intermediate node n to the end point. The cost function and the estimation function can have multiple setting schemes according to different problems.

Since the A* algorithm is a commonly used direct search algorithm, its principle is simple and efficient. Starting from the starting point, the A* algorithm will constantly iteratively update the cost function value of the adjacent point, and select the point with the smallest f(n) value as the next expansion point for path exploration to the end point. Under the A* algorithm, the planned path between the starting point to the end point is the optimal path under this heuristic function.

### 3.1. Grid Map Construction

**Figure 3:**
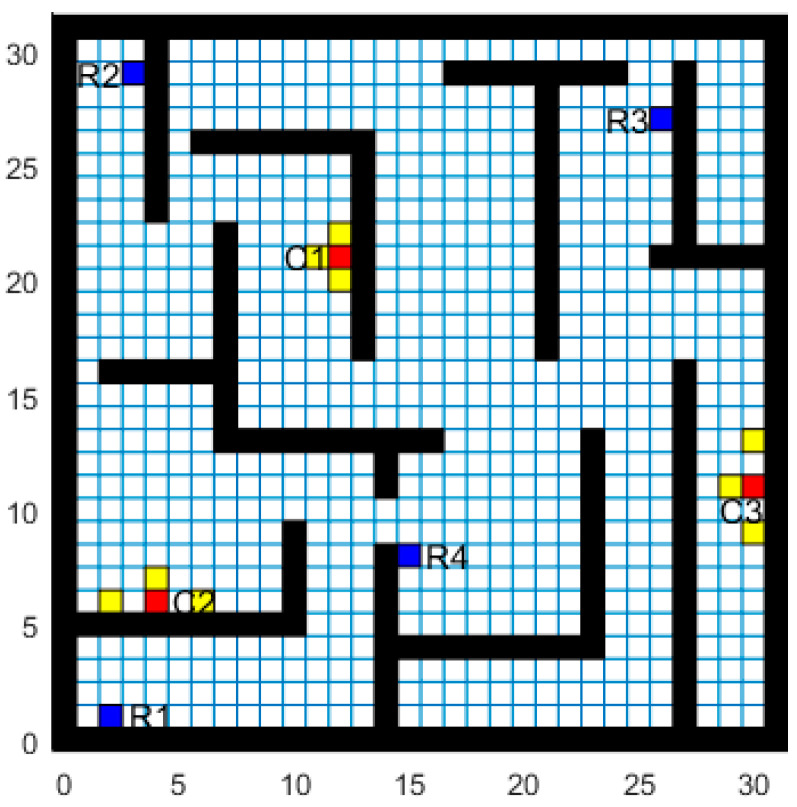
Grating diagram of the underwater environment.

Modeling the map environment with the grid method is a common classical graph algorithm in path planning research [23]. In this paper, the underwater environment was dispersed into grids of the same size and shape, and the size needed to satisfy the kinematic constraint of the AUV. The grid has only two states, namely “Free” and “Occupied” [18]. The simulation experiment environment based on the actual underwater obstacles was determined, as shown in Figure 3. The 2D grid environment range was set to the size of 30 m × 30 m, where the unit grid size was set to 1 m. Usually, there are static obstacles such as reefs and rock walls in the underwater environment, which are indicated by long black bars. In the deployed multi-AUVs data acquisition system, the AUVs, the task targets, and the underwater nodes are represented by blue, red, and yellow squares, respectively. In addition, the remaining white squares indicate the space where the AUV can travel freely underwater.

### 3.2. Path Planning

The traditional task allocation and path planning algorithm only considers the influence of the path length on the algorithm, but the steering angle of the AUV also needs to be considered. To further reduce energy consumption, the planned path of the AUV needs to be the shortest path and possess the minimum steering angle. Therefore, an AE algorithm that can further reduce energy consumption was proposed. The AE algorithm was also divided into the following two aspects: path planning and task assignment. The specific improvements of the former are as follows:

First, the heuristic function of the traditional A* algorithm was optimized to shorten the optimal path length. Heuristic functions usually set the generation value according to the distance, and generally there are several traditional distance algorithms, such as Manhattan distance and Euclidean distance. However, none of these algorithms consider the influence of diagonal distance on path planning. Therefore, the estimation function makes the following improvements:

(12) $$h\left(n\right)=\sqrt{2}\times n_{1}+n_{2},$$

where $\sqrt{2}\times n_{1}$ is the diagonal distance from the current node n to the target node g. $n_{2}$ is the horizontal–vertical straight distance from the current node n to the target node g. In the search of path points, the algorithm not only expands horizontally or vertically, but also considers the path points in the diagonal direction. Details can be derived using the following formula:

(13) $$n_{1}=\underset{}{\min}\left\{\left|x\left(n\right)-x\left(g\right),y\left(n\right)-y\left(g\right)\right|\right\},$$

(14) $$n_{2}=\underset{}{\max}\left\{\left|x\left(n\right)-x\left(g\right),y\left(n\right)-y\left(g\right)\right|\right\},$$

**Figure 4:**
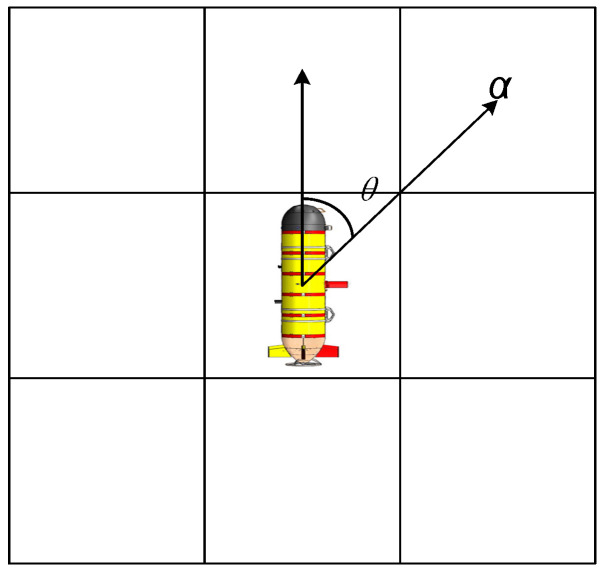
AUV steering schematic diagram.

Second, the steering cost is introduced to reduce the electric steering angle. According to the Chebyshev distance [24], when the AUV moves along the path, it can move forward to the eight adjacent points around it, as shown in Figure 4. It indicates the steering Angle of the AUV to the next path point. As seen in Equation (2), the AUV consumes energy when steering. Therefore, the steering cost needs to be increased in the planning path. Usually, the AUV turn of 90° is a one-unit distance, so the steering cost can be expressed as follows:

(15) α=2θ/π,

When the AUV steering at 45° enters the next path point, the cost function needs to add the steering cost amount to further constrain the steering angle of the AUV. In particular, the forward direction of the AUV starting point and the starting direction of the optimal path remain consistent to avoid interference with the path by the initial body position.

### 3.3. Task Assignment

The previous section described the improvement of AE algorithm path planning, leading to the following discussion on AUV task assignment. In Section 2, the relevant models of the hydro acoustic communication network were described, including nodes, tasks, cluster models of the AUV, and the corresponding energy consumption formula. Once the network task cluster $C=\left\{c_{1},c_{2},\ldots ,c_{k}\right\}$ is determined, it needs to assign the corresponding AUV from the AUV cluster $R=\left\{R_{1},R_{2},\ldots ,R_{m}\right\}\left(m\geq k\right)$. Through the AE algorithm, which is used to plan the task target path corresponding to all AUVs, and combined with Formulas (1)–(2), the cost matrix V of the required energy consumption composition of each $R_{j}\left(R_{j}\in R\right)$ corresponding to each task $c_{j}\left(c_{j}\in C\right)$ can be calculated. Finally, each task $c_{j}\left(c_{j}\in C\right)$ can be assigned the least energy and most constrained AUV under the constraint of Equations (3)–(6). The specific formula is as follows:

(16) $$V=\left[\begin{array}{cccc} v_{11} & v_{12} & \cdots & v_{1m}\\ v_{21} & v_{22} & \cdots & v_{2m}\\ \vdots & \vdots & \ddots & \vdots \\ v_{k1} & v_{k2} & \cdots & v_{km} \end{array}\right],$$

(17) $$v_{\mathrm{iimin}}=\underset{}{\min}\left\{v_{\mathrm{ij}},j=1,2,\ldots ,m\right\},$$

where *m* is the number of AUVs, and *k* is the number of targets. $v_{\mathrm{ij}}$ is the energy consumption cost of the j-th AUV corresponding to the i-th (i=1,2,…,k) target task. $v_{\mathrm{iimin}}$ indicates that the lowest energy cost of the i-th row of the matrix *V* is at the i-th (i = 1, 2,..., *k*) row and the imin-th (imin=1,2,…,m) column. That is, compared to other AUVs, the imin-th AUV has the lowest energy cost when performing the i-th target task and meets the above constraints. However, it should be noted that some target tasks may correspond to the same AUV. So, the new task allocation rules are formulated to avoid allocation conflict. In the first round of allocation, no conflicting target tasks are assigned to the least powerful AUV, and the remaining conflicting target tasks are temporarily not assigned to the AUV. In the second round of assignment, the first round of assignment is repeated for the remaining target tasks and AUVs. The system does not end until all target tasks are executed by the assigned AUV. The specific task assignment process is as follows:

1. Based on the path planning information and the energy consumption formula, the energy consumption cost of all AUVs performing each target task $\;v_{\mathrm{ij}}\left(i=1,2,\ldots ,k;j=1,2,\ldots ,m\right)$ can be calculated;
2. The first round of allocation: any two target tasks i and l are known. When i=1,2,…,k;l=1,2,…,k;i≠l, and imin≠lmin, then the imin-th AUV is the winning AUV of the i-th target, and the lmin-th AUV is the winning AUV of the l-th target. When i=1,2,…,k;l=1,2,…,k;i≠l, imin=lmin, and $v_{\mathrm{iimin}}<v_{\mathrm{llmin}}$, the imin-th AUV is the winning AUV of the *i*th target, and the l-th target is not assigned an AUV; if $v_{\mathrm{iimin}}>v_{\mathrm{llmin}}$, the lmin-th AUV is the winning AUV of the l-th target, and the i-th target is not assigned an AUV; if $v_{\mathrm{iimin}}=v_{\mathrm{llmin}}$, then the i-th target and the l-th target are temporarily not assigned AUVs. The second round of allocation: repeat the previous round of assignment in the remaining target tasks and unassigned AUV. The entire process is repeated until the corresponding AUV performs all the target tasks.

## 4. Simulation and Studies

### 4.1. Network Simulation Environment

The main purpose of this paper was to study the task assignment and path planning method of multi-AUVs data acquisition systems of underwater acoustic communication networks, so each AUV can be regarded as a particle without size and shape. In order to better compare the energy consumption per unit time of the algorithm, the energy efficiency ratio index $P_{t}$ was introduced to quantitatively analyze the energy efficiency performance of the multi-AUVs data acquisition system. The specific formula is as follows:

(18) $$P_{t}=\frac{E_{\mathrm{Total}}}{T},$$

where $E_{\mathrm{Total}}$ is the total energy consumed by the AUV in the cluster network. It contains two elements of energy consumption which are as follows: AUV motorized energy consumption $E_{\mathrm{Tri}}$ and the data of transmission energy consumption $P_{\mathrm{tx}}$. T is the total workload of AUV in the cluster network, approximated by the amount of data collected by the AUV.

To better evaluate the performance of the proposed algorithm, the BISOM algorithm [17] and the BINN algorithm [18] were added for comparison. In addition, the MATLAB R2021a software platform was used to perform simulation experiments, for which the main simulation parameters are listed in Table 1.

### 4.2. Results and Analysis

In Section 3.1, the underwater obstacle environment was represented on a two-dimensional grid map with four AUVs (R1, R2, R3, R4), three task targets (C1, C2, C3) and some underwater nodes. Usually, the cruise speed of the AUV is set to 6 m/s [19], but considering the small scope of the 2D grid environment in this project and the limited range of data acquisition, the cruise speed of the AUV was set to 3 m/s. Meanwhile, it should be noted that the energy consumption of the AUVs is solely the energy relative to absolute displacement. Considering the influence of water flow and other unknown factors, the unit energy consumption of the AUVs was approximated as 3 J/m to provide a reasonable safety margin. Moreover, the data modulation–demodulation between AUVs and underwater nodes is more complicated. The hydroacoustic communication modulation model in [25] was referenced to simplify the correlation model. Specific target task parameters are represented in Table 2, where a packet carries information in one unit of bits.

**Figure 5:**
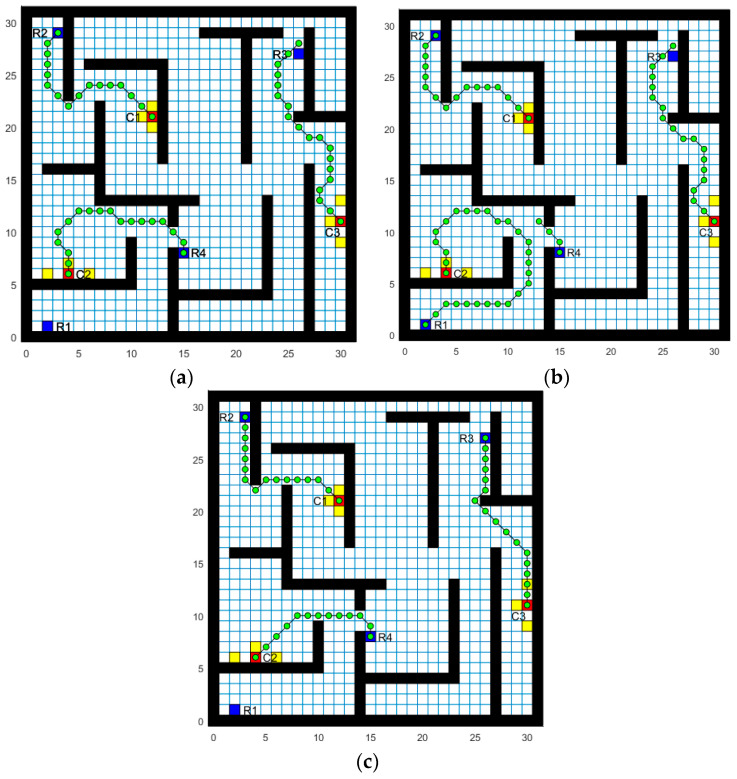
(**a**) Based on the BINN task assignment and path planning algorithm; (**b**) Based on the BISOM task assignment and path planning algorithm; (**c**) Based on the AE task assignment and path planning algorithm.

The BISOM, BINN and AE algorithms, respectively, performed simulation experiments in the simulation network constructed and represented in Figure 3. Figure 5a–c show the simulation results for the BISOM, BINN, and AE algorithms on task assignment and path planning, respectively. Since the task assignment only considers the Euclidean distance between the AUV and the target task, the BISOM algorithm assigns the AUV with the shortest Euclidean distance (R1) to the target task of C2, and also repeatedly assigns the AUV(R4), thus creating unnecessary paths. However, the BINN and AE algorithms consider the influence of underwater obstacles and rationalize them reasonably. As can be seen from Figure 5a,c, C1 was assigned to AUV (R2), C2 was assigned to AUV (R4), and C3 was assigned to AUV (R3). The AUV reached the target task without collisions and without repeated AUV assignments. The BINN algorithm is a mapping algorithm based on the biological incentive neural network. Its input excitation mainly includes the following two parts: the self-excitation of the target point the weighted excitation of surrounding neurons’ propagation, and the inhibitory input of obstacles. Due to the low number of propagating active neurons near the boundary and obstacles, the neuronal activity value at this place is generally low, and the resulting path is unreasonable. As shown in Figure 5a,c, in the path of R2–C1, R3–C3, near the disorder, the BINN algorithm tends to move towards the middle idle high viability zone. The irregular movement of the BINN algorithm not only generates redundant paths, but also increases the steering angle of the AUV. In addition, both the BINN and BISOM algorithms ignore the impact of the path angle on energy consumption, whereas the AE algorithm considers the AUV steering angle while ensuring the shortest path.

**Figure 6:**
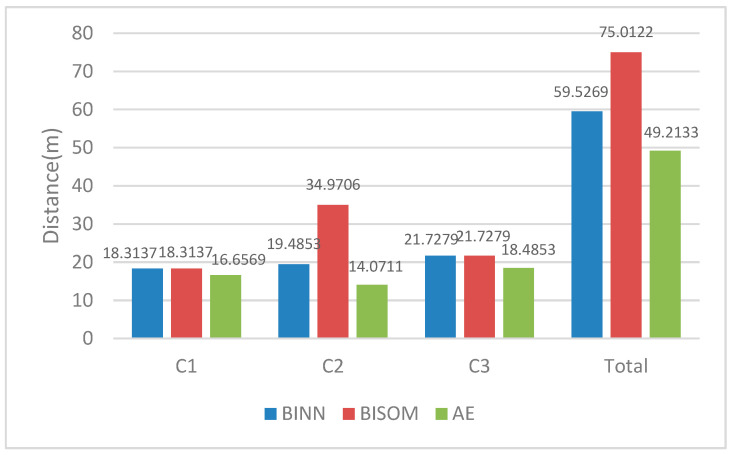
Total distance map of multi-AUVs data acquisition operation.

Figure 6 shows the actual navigation distance from the AUV to the targets. The sum navigating distances of the multi-AUVs system for C1, C2 and C3 are 59.5269 m, 75.0122 m and 49.2133 m in the BINN algorithm, the BISOM algorithm and the AE algorithm, respectively. Compared with the BINN and BISOM algorithms, the AE algorithm saved on 17.3% and 34.4% of the total navigation distance. In the BISOM algorithm, the task target C2 is significantly high due to unreasonable AUV (R1) and repeated AUV (R4) allocation. In the BINN algorithm, the generation of unnecessary paths also increases the planned path distance.

**Figure 7:**
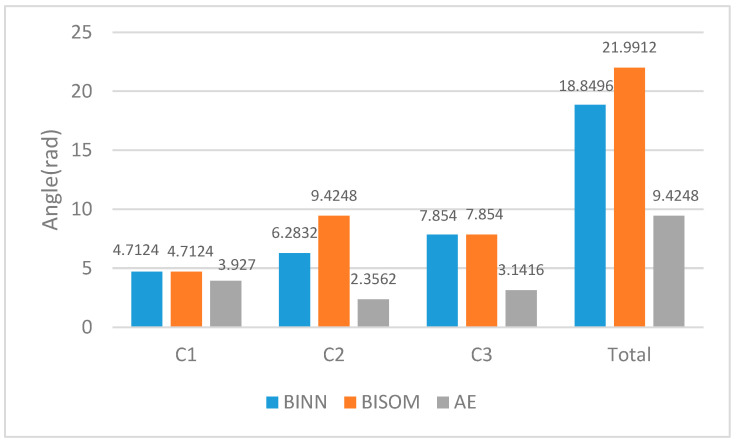
Actual navigation angle diagram of the AUV to the target location.

In addition to ensuring the shortest path, the steering maneuver angle of the AUV needs to be further reduced. As shown in Figure 7, the actual navigation total angles in the BINN, BISOM, and AE algorithms, of C1, C2, and C3, are 18.8496 rad, 21.9912 rad, and 9.4248 rad, respectively. Compared to the BINN and BISOM algorithms, the AE algorithm reduced the total navigation angle by 50% and 57.1%, greatly reducing the AUV motorized steering angle. The BINN and BISOM algorithms ignore the AUV steering angle. At the same time, due to the input inhibitory signal of the biological excitation neural network constantly changing, the AUV needs to constantly adjust its body position in the path and turn frequently, resulting in the increase in the total navigation angle.

**Figure 8:**
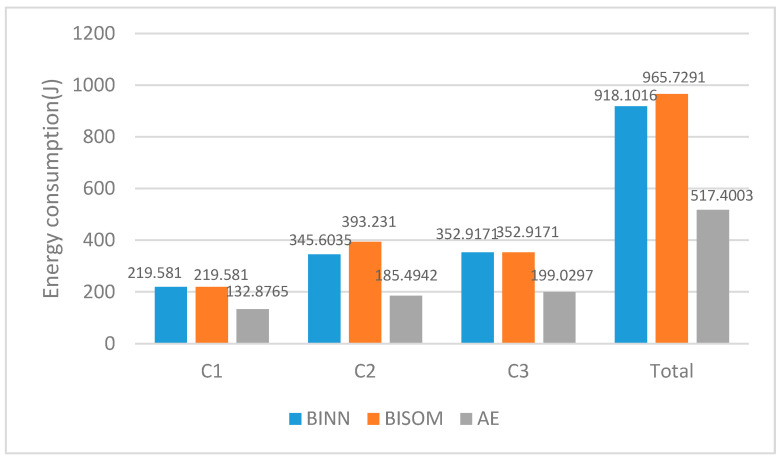
Actual energy consumption diagram of the AUV to the target location.

As shown in Figure 8, the mobile energy consumption and transmission energy consumption during the multi-AUVs data acquisition can be calculated by relevant formulas after the shortest path and minimum steering angle are guaranteed. In the BINN, BISOM, and AE algorithms, the actual energy expenditure of C1, C2, and C3 are 918.1016 J, 965.7291 J and 517.4003 J, respectively. Obviously, the AE algorithm compared with the BINN and BISOM algorithm reduced the total energy consumption by 43.6% and 46.4%, greatly reduced the energy consumption of AUV in the underwater environment, and extended the working time and life. Since BINN and BISOM algorithms do not use AUV as the cluster head strategy, the cluster data of these two algorithms needs to be transmitted to the cluster head node at the target task, and the AUV receives the collected data from the cluster head node at the target task, while the AE algorithm collects the data of the child nodes in the cluster directly at the target task.

**Figure 9:**
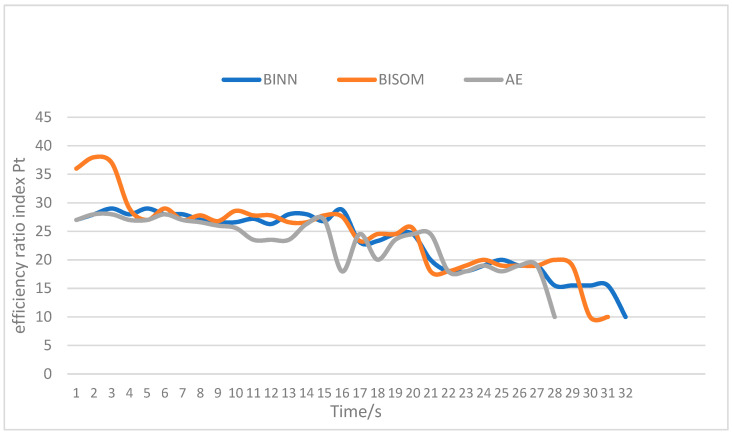
Index diagram of the energy efficiency ratio of multi-AUVs data acquisition operations.

In order to intuitively compare the energy efficiency of multi-AUVs data acquisition operations, the real-time energy efficiency ratio index $P_{t}$ was simulated on the whole multi-AUVs data acquisition system, and the energy consumption per unit time of AUV was recorded in the whole operation process, as shown in Figure 9. By optimizing the AE algorithm, the operation time of the whole AUV single data acquisition was reduced by about 12%. The overall energy efficiency ratio index $P_{t}$ of the AE algorithm was lower than that of the BINN and BISOM algorithms. This shows that the AE algorithm can maintain low energy consumption and high work efficiency in AUV data acquisition per unit time.

After many simulations, the proposed AE algorithm had more advantages over other algorithms in terms of energy consumption. Although the scope of the simulation environment, the number of AUVs and target tasks are limited, the algorithm can also be extended to more complex underwater obstacles, and even has important guiding significance for real underwater AUV data acquisition. Unlike other algorithms that can keep a safe distance from obstacles, the AE algorithm can find the lowest energy consumption path along the obstacle boundary and has the function of data collection, which are advantages of the algorithm.

## 5. Conclusions

In this article, a multi-AUVs collaborative optimization algorithm was proposed. In view of the energy consumption of multi-AUVs data acquisition systems in water acoustic cluster networks, the algorithm was optimized in the following three aspects: path planning, task assignment, and data acquisition. In path planning, the algorithm further optimized the path distance and steering angle based on the A* algorithm. In task assignment, the proposed algorithm suggested new allocation rules to solve the problem of the allocation conflict. In terms of data acquisition, the algorithm proposed the strategy of AUVs acting as the cluster head node, which further reduced the energy consumption of data transmission. In addition, simulation experiments show the effectiveness of the algorithm. Not only can all targets be accessed successfully, but also the total distance and total angle traveled by the AUVs system is reduced and the energy efficiency is improved. Otherwise, this approach can be extended to more complex 3D environments and adapted to more flexible scenarios. The errors, disconnections and failures of the algorithm are problems that we need to explore, classify, and solve in more detail in the future to solve the above environments and scenarios.

## Figures and Tables

**Table 1:** Control parameters.

Parameters	Value	Description
*S*		Underwater node
*C*		Target task
*R*		AUV
*energy*	15 kJ	AUV primary energy
*V_AUV_*	3 m/s	AUV cruising speed
*E_ti_*	3 J/m	AUV per unit of propulsion energy consumption
*E_turn_*		AUV steering is for energy consumption
*E_Tri_*		Total energy consumption
*d_0_*	10 m	Transmission threshold distance
*f*	1 kHz	Transmission frequency
*a*(*f*)		Absorption factor
*q_sp_*	1 mJ/bit	Energy consumption for data processing when sending
*q_rp_*	1 mJ/bit	Energy consumption for data processing upon reception
*E_ij_*		Unit energy consumption of data transmission
*E_tx_*		Total energy consumption of data transmission

**Table 2:** Target task parameters.

Target Task	Database	Corresponding Distance (m)
*C1*	10/20/50	1/1/1
*C2*	10/20/50	1/2/1
*C3*	10/20/50	1/2/1

## Data Availability

The data presented in this study are available on request from the corresponding author.
